# Clozapine: prevalence and modalities of associations with antipsychotics and mood stabilizer in French psychiatric hospitals. Multicenter survey on a given day

**DOI:** 10.1192/j.eurpsy.2025.770

**Published:** 2025-08-26

**Authors:** M. Bordes, R. Klein, E. Queuille

**Affiliations:** 1 Haute-Garonne, Ferrepsy, Toulouse; 2 Gironde, CH Charles Perrens, Bordeaux; 3 Nord, Association du réseau PIC, Armentieres, France

## Abstract

**Introduction:**

Clozapine is indicated for resistant schizophrenia as monotherapy. However, the response is inadequate in 40-70% of patients. In this context, the combination of clozapine with a second antipsychotic or a mood stabilizer is a strategy frequently used to potentiate its effects (**Barlatier, A. (2014)** *Human Medicine and Pathology.* [Doctoral thesis, University]). However, the level of evidence for these practices remains low, and data on the prevalence of such combinations in France are limited. Against this backdrop, collaboration between a national multi-professional network operating in various public and private mental health establishments (the PIC network) and a regional psychiatric research federation (FERREPSY Occitanie) enabled the study of the prevalence and modalities of these associations in a large panel of French psychiatric establishments.

**Objectives:**

Estimate the prevalence of co-prescriptions of antipsychotics and mood stabilizer with clozapine for patients hospitalized in full-time psychiatry.

**Methods:**

Observational cross-sectional study conducted on a given day in December 2023 in 30 participating centers that are members of the PIC network and/or FERREPSY.

**Results:**

The computerized records of 795 patients were analyzed by the referring pharmacists at the participating centers. 78.4% of patients had at least one antipsychotic in association with clozapine. 64.5% of antipsychotics associated with clozapine were conventional antipsychotics. Among atypical antipsychotics, aripiprazole was combined with clozapine in 9.9% of patients, amisulpride in 10.7%, risperidone in 8.2%, olanzapine in 4.3% and quetiapine in 3%. For mood stabilizer, the combination of clozapine with valproate was the most commonly used combination (23.64% of patients), ahead of lithium salts (15.6% of patients) and lamotrigine (10.1% of patients).

**Conclusions:**

The combination of psychotropic drugs with clozapine remains a majority practice, which seems to have little connection with existing literature data.

**Disclosure of Interest:**

None Declared

